# A Dual Stream Deep Learning Framework for Alzheimer’s Disease Detection Using MRI Sonification

**DOI:** 10.3390/jimaging12010046

**Published:** 2026-01-15

**Authors:** Nadia A. Mohsin, Mohammed H. Abdul Ameer

**Affiliations:** 1 Department of Computer Science, Faculty of Computer Science and Mathematics, University of Kufa, Najaf 54001, Iraq; 2 Department of Computer Science, Faculty of Education for Women, University of Kufa, Najaf 54001, Iraq; mohammed.almayali@uokufa.edu.iq

**Keywords:** Alzheimer disease, deep learning, MRI, sonification, multimodal

## Abstract

Alzheimer’s Disease (AD) is an advanced brain illness that affects millions of individuals across the world. It causes gradual damage to the brain cells, leading to memory loss and cognitive dysfunction. Although Magnetic Resonance Imaging (MRI) is widely used in AD diagnosis, the existing studies rely solely on the visual representations, leaving alternative features unexplored. The objective of this study is to explore whether MRI sonification can provide complementary diagnostic information when combined with conventional image-based methods. In this study, we propose a novel dual-stream multimodal framework that integrates 2D MRI slices with their corresponding audio representations. MRI images are transformed into audio signals using a multi-scale, multi-orientation Gabor filtering, followed by a Hilbert space-filling curve to preserve spatial locality. The image and sound modalities are processed using a lightweight CNN and YAMNet, respectively, then fused via logistic regression. The experimental results of the multimodal achieved the highest accuracy in distinguishing AD from Cognitively Normal (CN) subjects at 98.2%, 94% for AD vs. Mild Cognitive Impairment (MCI), and 93.2% for MCI vs. CN. This work provides a new perspective and highlights the potential of audio transformation of imaging data for feature extraction and classification.

## 1. Introduction

Alzheimer’s disease (AD) is a disorder that affects the brain cells and gradually demolishes memory, destroys thinking, and, over time, causes patients to lose the ability to perform basic daily tasks. In the early stages of AD, many changes occur in the brain, including the accumulation of proteins that form tau tangles and amyloid plaques. Eventually, healthy neurons will lose connection, stop working, and die. These changes may start a decade before the symptoms become noticeable. It damages the parts of the brain responsible for forming memories, the hippocampus and the entorhinal cortex. At the final stage of AD, the number of dying cells increases, and the damage significantly shrinks the brain tissue [1]. It is predicted that by 2025, over 1% of the population will be affected by AD or associated diseases. Most of these will require day-to-day care, depending on the disease’s progression, affecting the quality of life of patients and their families and placing enormous economic pressure [2].

Advances in neuroimaging have shown the potential of AD diagnoses with the aid of these modalities, such as magnetic resonance imaging (MRI), functional Magnetic Resonance Imaging (fMRI), computed tomography (CT), and positron emission tomography (PET). MRI images are a preferred choice for research and clinical screening due to their safety for repeated acquisition, lack of radioactivity, and their ability to provide high-resolution images [3,4]. However, relying solely on MRI images or visual Deep Learning (DL) models may miss minor information related to AD diagnosis. On the other hand, the multimodal structures that combine different data types such as images, speech, clinical assessments, and genetic information are often difficult to collect. This raises the need for alternative representations of the same data to reveal more hidden patterns [5].

DL methods, especially convolutional neural networks (CNNs), have achieved remarkable performance in AD research, as they can automatically extract relevant disease features instead of handcrafted ones [6]. CNNs have been widely investigated in the neuroimaging-based AD classification. These models have also been extended to other data modalities such as audio classification. However, a significant issue is the high computational requirements, especially when these networks become much deeper and more complex. Many limitations in the current literature, which can be summarized as follows:

- Invasive data modalities, some of the AD diagnosing models rely on imaging techniques that involve radioactivity, such as PET scans, which make them not suitable for long-term monitoring or repeated acquisition.
- Some multimodal approaches integrate heterogeneous data such as speech, clinical assessments, and genetic information, which are costly and hard to collect.
- Depending on computationally expensive architectures.
- Relying on a single data modality, such as MRI scans, which may fail in capturing latent AD-related features.

These limitations motivate the exploration of a lightweight multimodal approach that can extract complementary information while relying on safe and widely available MRI scans. The idea is to use complementary representations derived from the same MRI data, which may enrich the extracted features without the need for additional data sources.

Sonification, a technique that translates spatial and numerical data into non-verbal sounds that support information analysis, facilitate data interpretation, and lead to the discovery of latent data that may help in scientific, practical, or artistic purposes [7,8]. In medical imaging, sonification theory offers promising potential for assessing diagnoses by transforming spatial information into audio. For a complex and subtle progression disorder like AD, such an alternative representation can reveal additional disseminative features [5].

To effectively analyze the audio data, we adopted transfer learning, an efficient approach for feature extraction without the need for training deep models from scratch. YAMNet is a pretrained audio model based on the MobileNet-v1 architecture and trained on a large dataset of natural sounds. YAMNet produces three outputs per waveform: class scores, internal embeddings, and a Mel spectrogram [9].

In this study, we proposed a novel image-to-sound pipeline that is specifically designed to preserve anatomical structure while transforming selected MRI slices into audio waveforms. Our method employs a Gabor filter bank to extract rich texture features, followed by a Hilbert-space filling curve for the two-dimensional to one-dimensional mapping. The resulting sounds are directly processed by YAMNet, a pretrained audio model. To further enhance the system’s diagnostic accuracy, we trained a lightweight CNN model on the 2D MRI slices and fused the results of the two models. We can summarize the contribution of our work with the following main points:

- A novel MRI sonification framework that converts 2D MRI slices into audio signals using a Gabor filter bank and a locality preserving Hilbert curve.
- Using the Hilbert curve instead of simple raster row scanning for time mapping for locality preserving within the audio domain, which yielded better performance results.
- A dual-stream multimodal that learns from MRI slices via a lightweight CNN and their corresponding sonified audio representation through YAMNet embeddings, enabling cross-models feature enrichment for AD detection.

## 2. Related Work

Many studies have investigated DL approaches for Alzheimer’s disease detection, relying on neuroimaging data such as CT, PET, MRI, and fMRI. Most existing studies are either unimodal, using a single data modality, or multimodal, adopting heterogeneous data types such as different neuroimaging types, genetic information, or clinical data, which are often challenging to obtain, costly, and not routinely collected. However, some studies focused on generating other representations of the same data to catch underlying features [8]. In this study, we transform the selected 2D MRI slices into an audio form, enabling a multimodal framework that relies entirely on structural MRIs.

Ref. [10] proposed a deep learning approach named Adaptive Hybrid Attention Network (AHANet), which consists of two attention modules for local and global feature extraction from brain MRIs. The first is for extracting contextual and spatial data for long-term dependencies; the second is for capturing local features. The results demonstrated an accuracy of 98.53; however, achieving this required 75 training epochs and used 73,400 AD slices, 77,520 Mild Cognitive Impairment (MCI) slices, and 43,180 HC slices, which can be considered highly time- and resource-consuming.

Another study [11] proposed AlzheimerNet, a fine-tuned CNN designed to classify six classes, five AD stages, and one normal control. 23,805 MRI slices obtained from the ADNI dataset are used, achieving an accuracy of 98.67%. Their method relies heavily on extensive image augmentation to address data imbalance, yielding 60,000 images across the six classes, which can introduce certain dependencies and limitations [12].

The authors in [13] introduce a deep learning model consisting of two stages for automatic AD diagnosis, utilizing MRI images. They introduced an enhanced 3D DenseNet for brain tissue segmentation and an improved MobileNetV3 for classification. Their method achieved 97.85% classification accuracy in distinguishing AD from Cognitive Normal (CN). While demonstrating high accuracy, it relies solely on MRI images, without exploring other data formats.

Several studies investigated the use of PET scans for AD classification, as they provide valuable information regarding the disease. Deep learning and machine learning methods have been widely applied to PET data. For example, researchers in [14] introduced a multi-view separable pyramid network (MiSePyNet) that learns feature representations from multiple orientations of PET scans for AD classification. Their experiments yielded an accuracy of 83.05% for MCI prediction. Ref. [15] employed a wavelet transform as a fusion technique of MRI and PET scans to incorporate the metabolic and structural information. Researchers in [16] proposed a multimodal approach that combines heterogeneous features obtained from PET and MRI scans in a shared space where data from those modalities are mapped. This shared space reveals common patterns that help AD prediction. Another study [17] integrates MRI, PET, genetic data, and clinical scores to capture different aspects. Auto-Encoders used for feature extraction from clinical and genetic data while CNN-based model for feature extraction from imaging data. Although PET scans are valuable because they reflect cerebral metabolic activity and can reveal functional abnormalities earlier, they are expensive and require patients to receive radioactive tracers, making them less suitable for routine or repeated screening.

Another study [18] employed a convolutional autoencoder (CAE) to classify AD and MCI versus healthy controls using a single 2D MRI slice per subject from the OASIS dataset. Their method achieved an accuracy of 74.66, showing a moderate improvement over the basic 2D CNN. Researchers in [6] proposed a DANMLP, a multimodal system that integrates MRI, clinical, and genetic data. It combines a dual attention CNN with a multilayer perceptron (MLP) for feature extraction and classification of AD and MCI. They achieved accuracies of 93% for AD vs. MCI and 82.4% for MCI vs. CN. A CNN-based model known as PINet is introduced in [19]. The model takes MRI images as input along with privileged information (PI), a data driven from previous clinical findings. The PI is used to guide the model to the desired direction.

## 3. Materials and Methods

**Figure 1:**
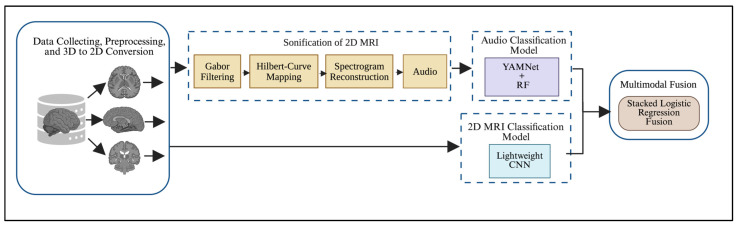
Research Workflow.

This section provides a detailed description of the dataset and the preprocessing steps, along with background on the methods used in this study. Figure 1 illustrates the overall framework, which begins with data acquisition and preprocessing, followed by the extraction of representative 2D slices from the 3D volume, and then a sonification pipeline for the selected 2D slices. The audio and 2D MRI slices are then classified using two models: YAMNet for audio and a lightweight CNN for images, followed by multimodal fusion for binary classification.

### 3.1. Alzheimer’s Disease Neuroimaging Initiative (ADNI) Dataset

The Alzheimer’s Disease Neuroimaging Initiative (ADNI) is an international project that supports research on AD. Sixty clinical sites across the USA and Canada are working on collecting the data to study the progression of the disease across normal, Mild Cognitive Impairment (MCI), and AD. Since access to this dataset requires permission, we applied to ADNI for access and received approval on 21 June 2023. A total of 3495 3D T1-weighted MRI scans were downloaded from the Image and Data Archive (IDA). The dataset was split on a subject level into 80% training, 10% validation, and 10% testing to balance sufficient training data with reliable and unbiased performance validation. The details of the downloaded 3D MRI images are described in Table 1. In this research, we did not adopt any augmentation technique since common transformations may distort some of the related disease structures and produce unrealistic variations [12].

### 3.2. 3D MRI Preprocessing

Initial preprocessing steps and the 2D slice extraction technique are explained in this section as follows

#### 3.2.1. Initial 3D MRI Preprocessing

**Figure 2:**
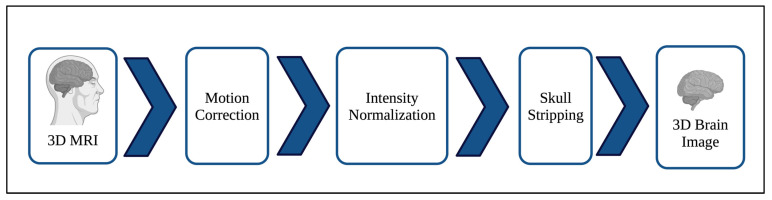
Preprocessing Pipeline.

Multiple preprocessing procedures are implemented to prepare the 3D MRI scans to ensure the best classification results. We utilized FreeSurfer (version 7.1.1), a well-known open-source tool specifically designed to process structural MRI images, to prepare images obtained from the ADNI dataset. Autorecon1 is applied to perform the essential procedures such as motion correction, skull stripping, and intensity normalization. Motion correction fixes the minor head movements that occur during the scan capture. The skull stripping process removes all the non-brain tissues, while the intensity normalization normalizes the MRI intensities to be in a standard range. Background removal is also implemented to ensure that only the brain images are obtained. Figure 2 demonstrates the initial preprocessing pipeline.

#### 3.2.2. 2D MRI Slice Selection

**Figure 3:**
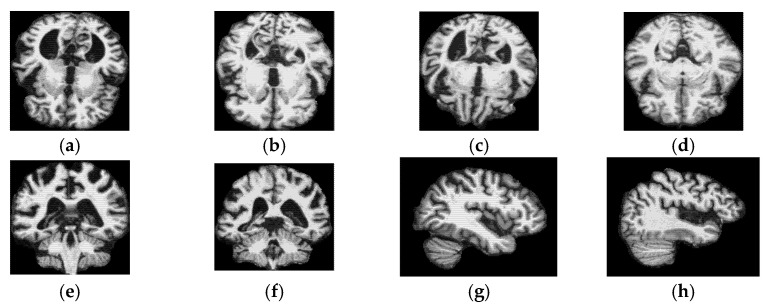
Samples of the selected 2D MRI slices. (**a**) AD—Segment 9; (**b**) CN—Segment 9; (**c**) AD—Segment 10; (**d**) CN—Segment 10; (**e**) AD—Segment 7; (**f**) MCI—Segment 7; (**g**) CN—Segment 4; (**h**) MCI—Segment 4.

Each preprocessed 3D MRI volume was reduced into a set of 2D MRI slices for the three main orientations, axial, coronal, and sagittal. A total of nine slices are extracted from each 3D MRI scan and treated separately in each experiment. The slice selection process follows the procedure described in our earlier publication. Within each predefined segment, the slice selection process was guided by a feature entropy, which is applied uniformly across all slices within each segment and across all orientations, to demonstrate specific slices that capture structural patterns and are highly informative for distinguishing Alzheimer’s disease [20]. Figure 3 displays a sample of the selected 2D MRI slices.

### 3.3. Methodological Background

#### 3.3.1. Multiple Scales and Multiple Orientations Gabor Filters

The Gabor filter is a linear filter whose impulse response is a sinusoidal wave convolved by a Gaussian envelope. It is widely used for texture feature analysis due to joint localization in the spatial and frequency domains. The two-dimensional complex Gabor filter with both of its components, real and imaginary, can be defined as in Equations (1)–(3) [21]:

(1) $$g\left(x,y;\theta ,f_{0}\right)=\frac{f^{2}}{\pi \gamma \eta }\exp\left(-\left(\frac{f^{2}}{\gamma^{2}}x^{\prime 2}+\frac{f^{2}}{\eta^{2}}y^{\prime 2}\right)\right)(\cos\left(2\pi fx^{\prime }\right)+\;jsin(2\pi fx^{\prime }))\;,\;$$

(2) $$x^{\prime }=\;x\cos\theta +y\sin\theta ,$$

(3) $$y^{\prime }=\;-\;x\sin\theta +y\cos\theta$$

where *x* and *y* refer to the spatial coordinates of an input image *I*(*x*, *y*), *θ* is the rotation angle of the filter, and $f_{0}$ is the center frequency, and γ and η are two constants that control the Gaussian envelope across the vertical and horizontal axes, respectively.

To extract texture features, an input image *I*(*x*, *y*) is convolved with a two-dimensional Gabor filter *g*(*x*, *y*) in order to get a complex Gabor response. To ensure adequate coverage of the spatial frequency, the image is filtered with a bank of Gabor filters with multiple orientations and scales [22]. The multiple scales filtering ensures sensitivity to the structure of varying spatial frequencies, while the multiple orientations ensure capturing the directionality and anisotropic properties.

In this context, the scale parameter σ and the number of orientations *K* are two factors added to the Gabor filter [21], as in Equation (4):

(4) $$g\left(x,y;f_{0},\sigma ,K\right)=\frac{f_{\sigma }^{2}}{\pi \gamma \eta }\exp\left(-\left(\frac{f_{\sigma }^{2}}{\gamma^{2}}x^{\prime 2}+\frac{f_{\sigma }^{2}}{\eta^{2}}y^{\prime 2}\right)\right)\;.\;exp\;\left(2\pi f_{\sigma }x^{\prime }\right)\;$$

(5) $$x^{\prime }=\;x\cos\theta_{k}+y\sin\theta_{k}$$

(6) $$y^{\prime }=\;-x\sin\theta_{k}+y{\cos\theta }_{k}$$

And the orientations are defined as:

(7) $$\theta_{k}=\;\frac{\pi k}{K},\;k=0,1\ldots ,K-1$$

The magnitude response of the Gabor kernel at scale σ and orientation $\theta_{k}$ is obtained by convolving the input image *I*(*x*, *y*) with the corresponding complex Gabor filter. This process yields a set of (σ × K) magnitude response maps, which form a multi-scale, multi-orientation representation of the input image.

#### 3.3.2. Hilbert’s Space-Filling Curve Time Mapping

The Hilbert curves are continuous space-filling curves that have been used in a wide range of scientific fields. It provides a systematic mapping from two-dimensional coordinates to a one-dimensional sequence. One of its key features is that it preserves spatial locality, so that points in two dimensions tend to map to neighboring positions along the one-dimensional curve. This feature makes the Hilbert curve an ideal choice when maintaining spatial relationships is crucial [23,24].

**Figure 4:**
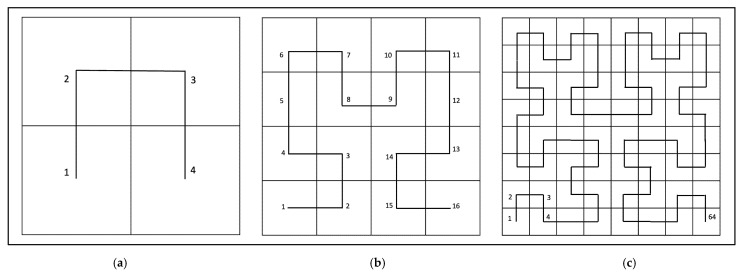
Hilbert’s space-filling curve at increasing orders, (**a**) order_1 (2 × 2 grid), (**b**) order_2 (4 × 4 grid), and (**c**) order_3 (8 × 8 grid).

The Hilbert curve visits every point in the *N* × *N* grid, where *N* = 2*^k^*, ensuring the recursive structure of the curve. Figure 4 illustrates the first three orders of Hilbert’s space-filling curve: (a) order_1 (2 × 2 grid), (b) order_2 (4 × 4 grid), and (c) order_3 (8 × 8 grid), showing how the traversal path covers the entire image [25].

### 3.4. The Proposed Methodology

In this section, we present an Alzheimer’s disease classification model dependent on structural MRI data. The proposed model introduces two complementary methods derived from the same underlying MRI slices. First, we proposed a sonification pipeline that transforms the 2D MRI slices into audio representations. Second, a dual-stream multimodal framework that integrates image and audio, resulting from the sonification phase, is used. By generating multiple forms of the same MRI data, the proposed methodology aims to capture distinct yet complementary features, and their joint modeling is used for better classification accuracy.

#### 3.4.1. Hilbert-Gabor-Based Sonification Pipeline

**Figure 5:**
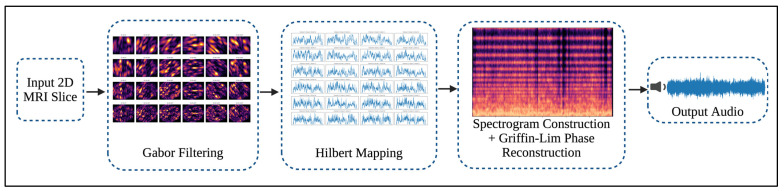
Hilbert-Gabor Sonification Pipeline.

In this subsection, we introduce a novel sonification pipeline that converts a 2D MRI image into an audio waveform, preserving both spatial and textural information. In addition to the conventional image classification approach, we introduced an alternative representation of the MRI slices. The objective of this step is to enable the use of the derived audio waveforms within the dual-stream multimodal framework. Figure 5 demonstrates the overall sonification pipeline.

The spatial textures in the 2D MRI images are encoded using a Gabor filter bank before converting them into audio signals. For the Gabor filter bank, we used four spatial scales and six orientations, yielding twenty four complex, distinct filters. The spatial frequencies were geometrically spaced so that lower frequencies (larger wavelengths) respond to broad anatomical regions, while higher frequencies detect fine details and boundary textures. The orientations (θ) ∈ {0, π/6, π/3, π/2, 2π/3, 5π/6} to provide a uniform directional coverage over 180°, ensuring sensitivity to structural patterns regardless of orientation. For each scale-orientation pair, the input 2D MRI image is convolved with the corresponding complex Gabor filter as defined in Equations (4)–(7). The magnitude of the complex response is then calculated as in Equation (8) to obtain the energy maps:

(8) $$E_{\sigma }{,}_{k}\left(x,y\right)=\;\left|I\left(x,y\right)*g(x,y;f_{\sigma },\sigma ,\theta_{k})\right|$$

The resulting twenty four energy maps retain information about textures across multiple scales and orientations, creating a set of feature maps that are later converted into time-varying signals.

Each Gabor energy map is transformed into a one-dimensional sequence using a Hilbert space-filling curve. Given the locality preserving property of the Hilbert curve described in Section 3.3.2, this mapping ensures that adjacent pixels in the two-dimensional image are still near each other in the resulting sequence. Since our images and their resulting Gabor maps may not be square or a power of 2, each energy map of size H × W is embedded into the smallest power of 2 grid to enable the construction of a Hilbert space- filling curve.

Using the Hilbert mapping function *h*(*d*), each index *d* in the one-dimensional space is mapped to a unique coordinate pair $\left(xd,\;yd\right)$ along the Hilbert traversal. Only the indices that satisfy the bounds x < W and y < H are retained as valid spatial coordinates. Let the total number of valid positions be *N_p_*, the Hilbert path *H* can be expressed as an ordered sequence of spatial coordinates:

$$H=[\left(x_{0},\;y_{0}\right),\;\left(x_{1}{,y}_{1}\right),\ldots \left(x_{N_{p}-1},y_{N_{p}-1}\;\right)]$$

To map the spatial Hilbert path into time frames, we divided it into *T* constative segments. The number of segments *T* is determined by the audio duration *T_sec_*, the sampling rate *SR*, and the hop size *h_hop_*, where each segment corresponds to one time frame in the resulting sequence. Each time frame *t* is associated with the contiguous set of spatial points of the Hilbert path. To turn all the Gabor energy maps corresponding to a scale-orientation pair $E_{\sigma ,k}$ into a time series, averaging over the spatial coordinates within each Hilbert segment as in Equation (9):

(9) $$e_{t}\left(\sigma ,\;k\right)=\frac{1}{\left|H_{t}\right|}\;\sum_{x,y\;\in {\;H}_{t}}E_{s,o}\left(x,y\right)$$

**Figure 6:**
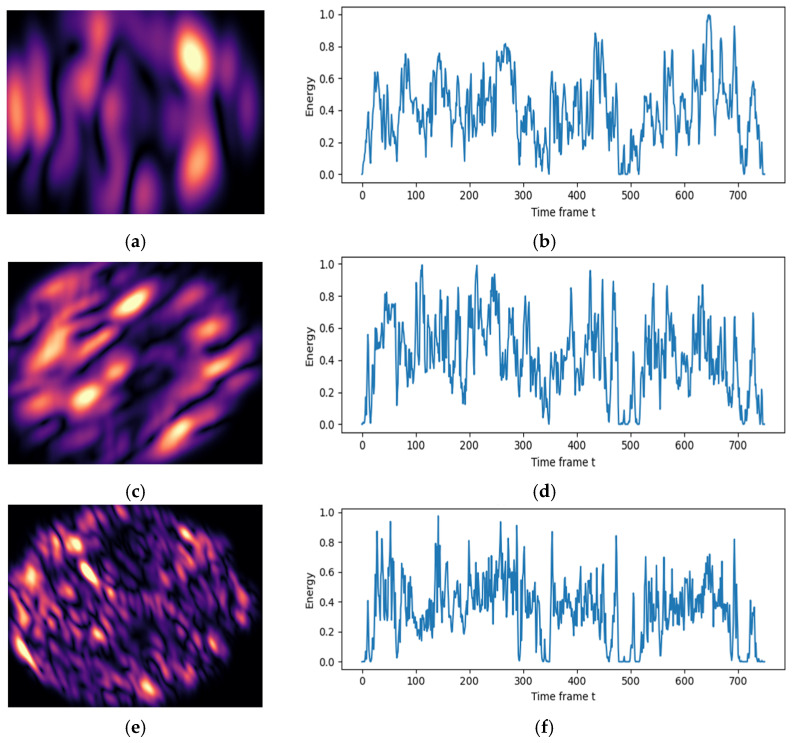
Example of Gabor energy maps with their corresponding Hilbert-based time series, (**a**) Energy map (scale 0, orient 0) (**b**) Energy time series channel 0, (**c**) Energy map (scale 1, orient 2) (**d**), Energy time series channel 8, (**e**) Energy map (scale 3, orient 5), and (**f**) Energy time series 23.

The output of the above steps is a set of one-dimensional time channels (one per Gabor channel). These sequences represent the energy fluctuation over time for a specific scale-orientation. Figure 6 shows a sample of Gabor energy maps with their corresponding Hilbert curve time series.

**Figure 7:**
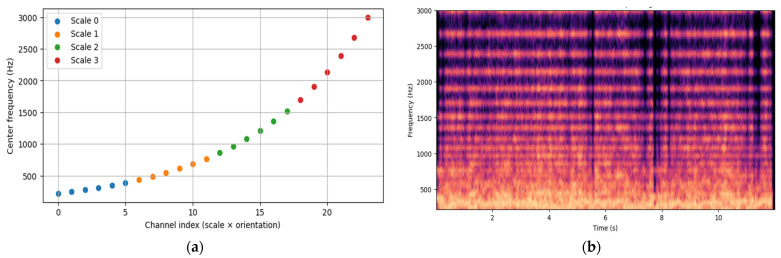
(**a**) Center frequencies assigned to the 24 Gabor channels, (**b**) Spectrogram Construction.

Following the Hilbert curve traversal, the twenty-four resulting time series are integrated into a unified time/frequency representation. The frequency band limit is defined as *f_min_* = 220 and *f_max_* = 3000 kHz. Geometrically, the set of time channels spans this frequency band such that each time series is placed into a distinct center frequency bin, as shown in Figure 7a. To construct the composite spectrogram, these time series are mapped to their respective frequency regions, as explained in Figure 7b. The lower-frequency Gabor features are assigned to the lower end of the band, while higher frequencies are assigned to the higher end.

The final magnitude spectrogram is converted to an audible waveform using the Griffin-Lim reconstruction. The Griffin-Lim estimates a phase signal that best matches the imposed magnitude pattern. The resulting 16 kHz audio is a single, continuous auditory representation of the 2D MRI slice. Algorithm 1 presents a pseudocode of the sonification pipeline.
**Algorithm 1:** Pseudocode of MRI to Audio Sonification Pipeline Input:       •2D MRI slice *I* of size *H* × *W*      •Target Duration *T_audio_*      •Sampling rate *f_s_* = 16 kHz      •Number of Scales *S*, Orientations *O*      •Frequency Range [*f_min_*, *f_max_*]      •Hop size Hop      •Number Griffin-Lim iterations *N_GL_*Output:
      •Audio waveform YStep 1: Image Loading and Normalization:
       1.I← Load 2D MRI slice       2.$I\leftarrow Normalize\left(I\right)\;to\;[0,1]$Step 2: Initialize Gabor Filter Bank *G*:       3.G←Ø       4.for s←1 to S do       5.        for o←1 to O do       6.                 $G\left(s,o\right)\leftarrow Construct\;complex\;Gabor\;filter$       7.                 $G\;\leftarrow G\cup \{G\left(s,o\right)\}$       8.        end for       9.end forStep 3: Gabor Energy Map Extraction E:       10.$for\;each\;G\left(s,o\right)\in G\;do$       11.       $R_{real}\leftarrow Convolve(I,\;Re\left\{G\left(s,o\right)\right\})$       12.       $R_{imag}\leftarrow Convolve(I,\;Im\left\{G\left(s,o\right)\right\})$       13.       $R\left(s,o\right)\leftarrow R_{real}+\;i\cdot R_{imag}$       14.       $E(s,o)\leftarrow \left|R(s,o)\right|$       15.end forStep 4: Hilbert curve time mapping       16.N ←Next power of 2≥max(H,W)       17.$H_{curve}\leftarrow Generate\;Hilbert\;curve\;on\;N\times N\;grid$       18.$H_{valid}\leftarrow Retrieve\;indices\;within\;H\times W$       19.$T\;\leftarrow ceil(T_{audio\;}\times \;f_{s}/Hop)$Step 5: Feature projection into spectrogram       20.C ←S×O Number of feature channels       21.$S_{mag}\leftarrow \;0$       22.for c←1 to C do       23.     $e_{c}\leftarrow Extract\;E\;values\;along\;H_{valid}\;segments$       24.     $e_{c}\leftarrow Avg(e_{c})\;within\;each\;segment$       25.     $f_{c}\leftarrow Assign\;center\;frequency\;in\;\left[f_{min},\;f_{max}\right]$       26.     $b_{c}\leftarrow Nearest\;frequency\;bin\;to\;f_{c}$       27.     $S_{mag}\left(b_{c}\;,\;:\right)\leftarrow S_{mag}\;\left(b_{c}\;,\;:\right)+e_{c}$       28.end forStep 6: Frame Normalization       29.$S_{mag}\leftarrow Normalize\left(S_{mag}\right)\;to\;[0,1]$ Step 7: Phase reconstruction (Griffin-Lim)       30.Φ←Random phase initialization       31.$for\;i\leftarrow 1\;to\;N_{GL}\;do$       32.     $y\;\leftarrow ISTFT(S_{mag},\;\Phi )$       33.    Φ ←Phase (STFT(y))       34.end for       35.$y_{raw}\leftarrow y$Step 8: Post Processing        36.$Y\leftarrow Normalize\;peak\;amplitude\;of\;y_{raw}$       37.return YComputational cost: $O\;(S\times O\;Gabor\;Convoluton+N_{GL}Griffin-Lim\;Iterration)$


#### 3.4.2. Dual-Stream Alzheimer’s Disease Classification Model

**Figure 8:**
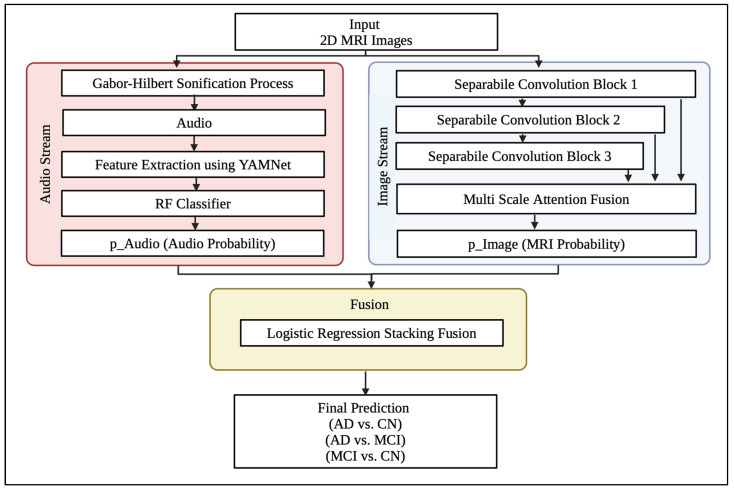
Dual-Stream Alzheimer Disease Classification Model.

Our proposed framework integrates structural brain MRI analysis with audio representations. The workflow consists of two complementary streams: an audio stream that employs YAMNet, a pretrained audio classification model, and a vision stream that processes individual 2D MRI slices using a lightweight CNN. These two modalities are then fused to improve the robustness and discriminative power of the Alzheimer’s disease detection. Figure 8 illustrates this dual-stream framework.

##### Audio Stream

The audio branch of our proposed model operated on sonified MRI slices, with each slice corresponding to a .wav file generated by the sonification pipeline. The audios yield a 16 kHz mono signal compatible with YAMNet input.

We used YAMNet as a feature extractor with pretrained weights kept frozen. For each audio signal passed to YAMNet, the network outputs a frame-level embedding. Each frame corresponds to a short audio window, represented as a 1024-dimensional vector, which are the learned features. The embeddings are then flattened and used as input vectors to a Random Forest (RF) classifier with 100 trees. The RF outputs the probability of Alzheimer’s disease based solely on the derived audio.

##### Image Stream

**Figure 9:**
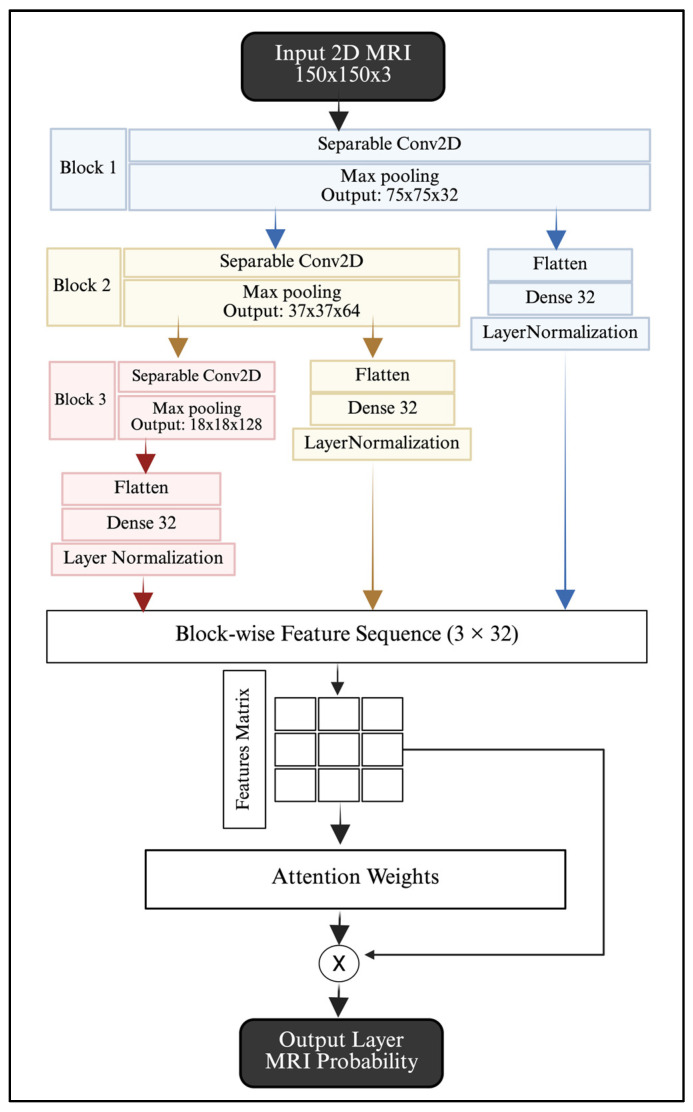
Image Stream Architecture.

The image branch processes the selected MRI slices using a lightweight CNN. We followed the architecture introduced in our earlier publication [20]. The network architecture consists of three depthwise separable convolutional blocks. Increasing kernel sizes are used at 32, 64, and 128, followed by a ReLU activation function and max pooling. The feature maps are extracted at different depths and then aggregated using an attention multi-level fusion. The final output of this model is a class probability representing the MRI prediction. Figure 9 and Table 2 illustrate the image branch details.

##### Multimodal Fusion

To combine information from the MRI and audio models, we adopt a stacked generalization framework using a logistic regression as a meta-classifier [26]. The MRI and audio streams are trained separately, where each model produces a probability of AD. Fusion strategy is performed on the probability level rather than latent feature representations, by stacking the MRI-based probability and the corresponding audio probability into a single feature vector to ensure feature alignment. For $x_{i}^{MRI}\;and\;x_{i}^{Audio}$ denote the MRI slice and the corresponding sonified audio signal for subject *i*, respectively. The image stream model $f_{MRI}$ and the audio stream model $f_{Audio}$ each produce a probabilistic prediction given by

$$p_{i}^{MRI}=f_{MRI}\left(x_{i}^{MRI}\right),\;\;and{\;p}_{i}^{Audio}=f_{Audio}\left(x_{i}^{Audio}\right)$$

where $p_{i}^{MRI}\;and\;p_{i}^{Audio}\in \left[0,\;1\right]$.

To prevent data leakage, out-of-fold (OOF) predictions are generated using the stratified K-fold cross validation. For each subject *i*, we obtained OOF estimates ${\hat{p}}_{i}^{MRI}\;and\;{\hat{p}}_{i}^{Audio}$ from the base models trained without using that subject. These predictions are stacked together in a two-dimensional vector as follows:

$$z_{i}=\left[\begin{array}{c} {\hat{p}}_{i}^{MRI}\\ {\hat{p}}_{i}^{Audio} \end{array}\right]$$

A logistic regression model is then trained on the stacked OOF vectors $\left\{z_{i}\right\}$ to learn the fusion function, as in Equation (10):

(10) $$p_{i}^{Fusion}=\;\sigma \;\left(w^{T}z_{i}+b\right)$$

where w and b are learned parameters and $\sigma \left(\cdot \right)$ denotes the sigmoid function. The resulting $p_{i}^{Fusion}$ represents the final multimodal probability estimate. of the disease. During model testing, the trained MRI and audio models produce $p^{MRI\;}and\;p^{Audio}$ for each test sample, which are stacked into $z_{test}=\left[\begin{array}{c} p^{MRI}\\ p^{Audio} \end{array}\right]$ and passed through the trained logistic regression meta-classifier to obtain the final prediction. Algorithm 2 summarizes the complete dual-stream framework.
**Algorithm 2:** Pseudocode of Dual-Stream Multimodal for Alzheimer’s Disease ClassificationInput:      •$2D\;\mathrm{MRI}\;\mathrm{slices}\;x_{i}^{MRI}$      •$\mathrm{Corresponding}\;\mathrm{audio}\;\mathrm{signal}\;x_{i}^{Audio}$      •$\mathrm{Ground}\;\mathrm{truth}\;\mathrm{labels}\;y_{i}$      •Number of folds K      •$\mathrm{Image}\;\mathrm{classifier}\;f_{MRI}\left(\cdot \right)$      •$\mathrm{Audio}\;\mathrm{classifier}\;f_{Audio}(\cdot )$      •Meta classifier g(·)Output:       •$\mathrm{Disease}\;\mathrm{probability}\;p_{i}^{Fusion}$Step 1: Data initialization      1.$D\leftarrow \left\{x_{i}^{MRI},\;x_{i}^{Audio},\;y_{i}\right\}\;$ load paired samples      2.${\hat{p}}^{MRI}\leftarrow 0,\;{\hat{p}}^{Audio}\leftarrow 0$ Initialize OOF vectorsStep 2: Stratified K-fold partitioning      3.F ←split D into K stratified foldsStep 3: OOF based model training      4.for k ← 1 to *K* do      5.      $f_{MRI}^{k}\leftarrow$ train image model on fold *k*      6.      $f_{Audio}^{k}\leftarrow$ train audio stream model on fold *k*      7.    $\;\mathrm{for}\;\mathrm{each}\;\mathrm{validation}\;\mathrm{sample}\;i\;\in \;F_{k}$ do      8.             ${\hat{p}}_{i}^{MRI}\leftarrow f_{MRI}^{k}(x_{i}^{MRI})$      9.             ${\hat{p}}_{i}^{Audio}\leftarrow f_{Audio}^{k}\left(x_{i}^{Audio}\right)$
      10.    end for      11.end forStep 4: Stacked feature construction      12.$z_{i}\leftarrow \;\left[{\hat{p}}_{i}^{MRI},{\hat{p}}_{i}^{Audio}\;\right]$Step 5: Multimodal fusion learning      13.g ←$\;\mathrm{train}\;\mathrm{logistic}\;\mathrm{regression}\;\mathrm{on}\;\left\{z_{i},y_{i}\right\}$Step 6: Model testing using the final image and audio models      14.$p_{i}^{MRI}\;\leftarrow f_{MRI}(x_{i}^{MRI})$      15.$p_{i}^{Audio}\;\leftarrow f_{Audio}(x_{i}^{Audio})$      16.$z_{i}^{test}\;\leftarrow [p_{i}^{MRI},\;p_{i}^{Audio}]$      17.$p_{i}^{Fusion}\leftarrow g(z_{i}^{test})$
$\mathrm{Return}\;p_{i}^{Fusion}$Complexity Cost: $O(K\cdot (train(f_{MRI})+train\;(f_{Audio}))+N)$


## 4. Experimental Results

### 4.1. Experimental Environment and Training Settings

All of our experiments are applied on a local machine (MacBook Pro, Apple Inc., Cupertino, CA, USA) equipped with an M1 chip, 16 GB of memory, and 10 cores running macOS Sonoma. Python 3.7.11 version is used as the programming language via anaconda navigator.

The lightweight CNN, MRI branch, is trained with the Adam optimizer, a learning rate of 0.0001, 20 epochs, a binary cross-entropy loss function, and a batch size of 32. For the audio branch, the pretrained YAMNet is used to extract features on 16 kHz audio, and the features are passed to an RF classifier with 100 trees. Out-of-fold predictions from the MRI and audio branches were utilized to train a logistic regression model for the final multimodal classification.

A K-fold stratified cross-validation strategy with F = 5 was applied, with 10% of the data held out for testing in each fold.

### 4.2. Evaluation Metrics

For each classification task, AD vs. CN, AD vs. MCI, and MCI vs. CN, the performance metrics are defined using four basic concepts: true positive (TP), false positive (FP), true negative (TN), and false negative (FN), and it is important to understand them in the context of this study. TP appears when the patient is correctly classified as the disease class of interest, AD or MCI, while the TN corresponds to the true identification of the non-disease class. FP occurs when a healthy subject is classified as diseased, on the other hand, the FN arises when a patient is falsely identified as non-diseased.

The performance of the proposed multimodal was assessed based on six standard performance metrics: accuracy, precision, sensitivity, specificity, F1 score, and Area Under the Curve (AUC). Equations (11)–(15) define the first five metrics, while AUC evaluates the model by plotting the TP Rate (TPR) to the FP Rate (FPR) [20].

(11) $$Accuracy=\;\frac{TP+TN}{TP+TN+FP+FN}$$

(12) $$Precision=\;\frac{TP}{TP+FP}$$

(13) $$Sensitivity=\;\frac{TP}{TP+FN}\;$$

(14) $$Specificity=\;\frac{TN}{TN+FP}\;$$

(15) $$F1=\;2\;\times \;\frac{precision\;\times \;sensitivity}{precision+sensitivity}$$

### 4.3. Results Discussion

This section presents the evaluation of the proposed sonification model and the dual stream classification framework. Two primary analyses are conducted. First, we assess the performance of the proposed Hilbert-based sonification by comparing it against a raster row mapping. Second, we evaluate the proposed dual-stream model through a comprehensive comparison of the selected 2D MRI slices with the model proposed in [20] and some baseline models. The experiments are conducted on images extracted from brain segments 4–12 across the axial, coronal, and sagittal orientations. Three classification tasks are considered: AD vs. CN, AD vs. MCI, and MCI vs. CN.

For the first performance analysis, Hilbert against raster row sonification, we applied the same sonification steps to the selected slices, except that the Hilbert-curve mapping was replaced with a row raster traversal, in which the 2D MRI is scanned row by row sequentially to construct the corresponding one-dimensional time series. This experiment evaluates the importance of spatial locality preservation in the audio transformation process. As summarized in Table 3, the Hilbert based audio representation clearly outperforms the simple raster row sonification across all segments, orientations, and classification tasks. This improvement is particularly in the AD vs. CN and AD vs. MCI tasks in coronal and axial planes. In the sagittal orientation, the raster row yielded comparable results in limited cases in AD vs. CN.

**Figure 10:**
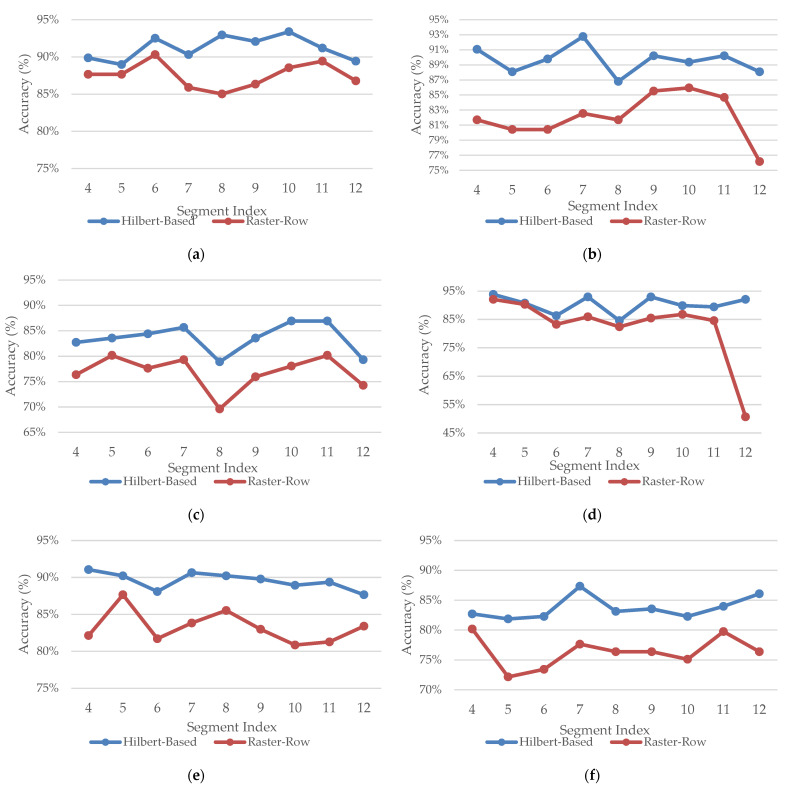
Segment-wise classification accuracy (%) comparison between Hilbert-based and raster-row sonification across axial and coronal orientations. Subfigures show results for (**a**) AD vs. CN (Axial), (**b**) AD vs. MCI (Axial), (**c**) MCI vs. CN (Axial), (**d**) AD vs. CN (Coronal), (**e**) AD vs. MCI (Coronal), and (**f**) MCI vs. CN (Coronal). Accuracy values are reported for individual selected 2D MRI slices from segments (Seg 4–12).

Figure 10 presents a detailed segment-wise comparison of classification accuracy yielded by applying Hilbert-based and raster-row sonification across different classification tasks and orientations. The Hilbert-based sonification approach consistently achieved higher accuracy than raster-row traversal across most segments. This demonstrates the benefit of preserving spatial locality during the conversion from 2D to 1D for AD detection. Even for the challenging MCI vs. CN classification task, where the overall performance is lower, the Hilbert audio maintained a clear advantage over the raster row sonification. These results confirm the importance of locality preserving traversal for capturing more discriminative audio representations for different anatomical segments.

Second, we assess the contribution of the dual-stream model and compare its performance with the lightweight CNN proposed in [20] that follows a similar architecture to the model introduced in the image stream section. More baseline models are included in the comparison, including a standard three-layer CNN, MobileNetV1 (MN-V1), and MobileNetV2 (MN-V2). To ensure a fair assessment, all the baseline methods were trained and evaluated using the same experimental setting mentioned in Section 4.1 (Experimental Environment and Training Settings).

The standard CNN is built from three convolutional blocks having 32, 64, and 128 filters, respectively, each followed by a max-pooling layer. The convolutional layer used a 3 × 3 kernel and a ReLU activation. MobileNetV1 and MobileNetV2 are used as fixed feature extractors, with their original classification heads removed. For all the baseline models, extracted feature maps are flattened and passed through a fully connected layer with 64 neurons, followed by a final sigmoid output classification layer.

**Figure 11:**
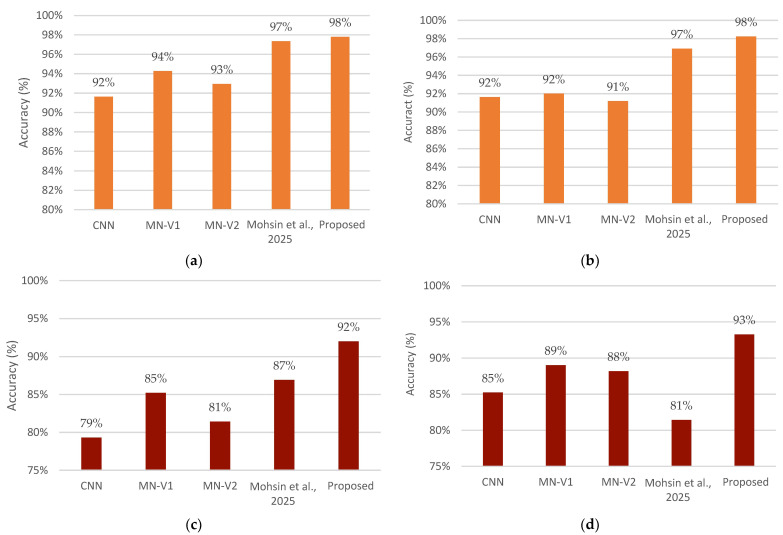

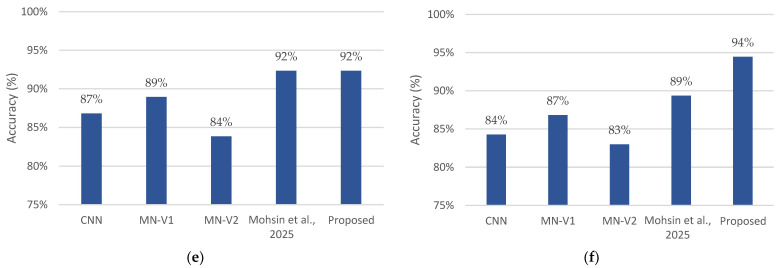
Accuracy comparison of the proposed dual-stream model and baseline architectures for selected best performance segments. (**a**) AD vs. CN (Seg 9, Axial), (**b**) AD vs. CN (Seg 10, Sagittal), (**c**) MCI vs. CN (Seg 4, sagittal), (**d**) MCI vs. CN (Seg 7, Sagittal), (**e**) AD vs. MCI (Seg 7, coronal), and (**f**) AD vs. MCI (Seg 10, sagittal). Mohsin et al. 2025 refers to [20].

Figure 11 presents a comparative accuracy analysis among the proposed dual-stream model, with the baseline models using carefully selected slices. The reason behind choosing these slices from these specific segments is to ensure a fair and unbiased comparison. We first extensively evaluated the model in [20] on all selected slices across segments 4–12, individually for each classification task and image orientation. For each task-orientation pair, segments that achieved the highest classification accuracy were selected. The corresponding results are reported in subfigures (a) AD vs. CN (Seg 9, axial), (c) MCI vs. CN (Seg 4, sagittal), and (e) AD vs. MCI (Seg 7, coronal). Subsequently, an identical procedure was applied to our model, which was also evaluated across all segments, and the slices yielding the highest accuracy were selected for comparison with the lightweight CNN model, as in Figure 11: (b) AD vs. CN (Seg 10, sagittal), (d) MCI vs. CN (Seg 7, sagittal), and (f) AD vs. MCI (Seg 10, sagittal).

Across all classification tasks and orientations shown in Figure 11 subplots, the proposed model consistently achieves the highest results, comparable to the other baseline models. The most noticeable improvement is seen in the MCI vs. CN, which is considered the most challenging classification task. However, Seg-7 in the sagittal plane showed competitive results in distinguishing AD from MCI between the dual-stream and the model proposed in [20].

A comprehensive comparison between the proposed model and the model reported in [20] is conducted, including accuracy, precision, sensitivity, F1 score, specificity, and AUC, and is presented in Table 4, Table 5 and Table 6. These tables report segment-wise performance across axial, coronal, and sagittal orientations for the three classification tasks.

Table 4 illustrates the results of AD vs. MCI. First, in the axial plane, the proposed model improved accuracy across all segments, with an increment from baseline values as low as 76.6% to a maximum of 91.5% at segment 4. Similar improvements are obtained in the sagittal plane, where the proposed model achieves its highest accuracy at 94.5%. In the coronal plane, the proposed method substantially enhances the performance even in challenging segments such as segment 8 (69.4% to 90.6%).

Across all orientations, the proposed dual-stream model also demonstrated consistent improvements in sensitivity alongside precision, F1-score, specificity, and AUC.

A detailed comparison of the MCI vs. CN classification task is presented in Table 5. Overall, the dual-stream model also records better performance results across all tasks and orientations, with consistent gains for all used performance metrics. The highest improvements occur when using the selected slices from segment 11 on the axial plane, and segments 4 and 5 on the coronal plane. The best accuracy is recorded in the sagittal plane of segment 7, yielding 93.2%.

Table 6 presents the comparative results for AD vs. CN, also for all segments and orientations. The results report a slight improvement in accuracy, yielding 98.2% for the selected slice from segment 10 in the sagittal plane. Although the dual-stream model achieved better accuracy in most segments, we can notice that segments 6 on sagittal, 5 and 7 on axial, and 10 on coronal have similar or slightly better accuracy.

Finally, we provided a computational complexity analysis of the multimodal, focusing on the MRI-based model since it is the trainable part of the multimodal. The audio stream relies on a pretrained model (YAMNet), which produced relatively low complexity. Table 7. Provided a comparative analysis of the MRI-based stream with three baseline lightweight architectures from highest to lowest: MobileNet1, EfficientNetB0, and MobileNetV2. It illustrates the number of floating-point operations, where the proposed method shows the lowest FLOPs, reflecting a significant reduction in computational complexity.

## 5. Conclusions

In this study, we introduced a dual-stream deep learning model that integrates 2D MRI information with a novel Hilbert-based sonification pipeline to improve the automatic Alzheimer’s disease detection using non-invasive neuroimaging. We investigated whether a complementary representation derived from the same neuroimaging data can improve Alzheimer’s disease classification. Features extracted from YAMNeT were combined with the features extracted from a lightweight CNN trained directly on MRI images, enabling multimodal fusion without requiring additional data modalities.

Comprehensive experiments across the three main orientations and nine anatomical segments showed that our model consistently outperforms baseline models for the AD vs. MCI and MCI vs. CN classification tasks, especially in segments where the baseline achieved relatively low accuracy. On the other hand, results for the AD vs. CN task showed a lower improvement rate due to the already high baseline performance, yet the proposed model maintained strong performance, achieving the highest accuracy of 98.2% at segment 10 in the sagittal plane. Despite these encouraging results, our study relies on a single dataset (ADNI), and although an extensive segment-wise and orientation-wise evaluation was conducted, the potential of dataset bias cannot be eliminated.

The achieved results demonstrate that the use of sonification offers meaningful additional information about MRI images and can be used as a supplementary input in a multimodal pipeline, raising the potential for early diagnosis of Alzheimer’s disease.

## 6. Future Work

Future work will explore extensions to this pipeline, such as a 3D image sonification pipeline, adapt the Hilbert curve to fit more in our framework, and evaluate the system using larger and different datasets. Furthermore, future studies will investigate the robustness of the proposed model regarding other dataset split configurations and assess the applicability of the proposed sonification-based methodology to other neurological disorders, such as brain tumors and intracerebral hemorrhage conditions, as well as cases where these instances occur with Alzheimer’s disease, in order to evaluate the method’s generalizability.

## Figures and Tables

**Table 1:** Details of 3D MRI scans.

Classes	Number of 3D MRI Scans	Training	Validation	Testing
AD	1125	900	112	113
CN	1146	916	115	115
MCI	1224	979	122	123

**Table 2:** The Architecture of the Image Stream.

Layer Type	Output Shape	Parameters Number	Connected to
Input Layer	150×150×3	0	-
Sep_Conv2D_B1	150 × 150 ×32	155	Input Layer
Max_Pooling_B1	75×75×32	0	Sep_Conv2D_B1
Sep_Conv2D_B2	75×75×64	2400	Max_Pooling_B1
Max_Pooling_B2	37×37×64	0	Sep_Conv2D_B2
Sep_Conv2D_B3	37×37×128	8896	Max_Pooling_B2
Max_Pooling_B3	18×18×128	0	Sep_Conv2D_B3
Flatten_B1	180,000	0	Max_Pooling_B1
Flatten_B2	87,616	0	Max_Pooling_B2
Flatten_B3	41,472	0	Max_Pooling_B3
Dense_B1	32	5,760,032	Flatten_B1
Dense_B2	32	2,803,744	Flatten_B2
Dense_B3	32	1,327,136	Flatten_B3
Layer_Normalization_B1 (N1)	32	64	Dense_B1
Layer_Normalization_B2 (N2)	32	64	Dense_B2
Layer_Normalization_B3 (N3)	32	64	Dense_B3
Stacked	3×32	0	N1, N2, N3
TimeDistributed Dense (32, ReLU) (TD_1)	3×32	1056	Stacked
TimeDistributed Dense (1, Linear) (TD_2)	3×1	33	TD_1
Attention Weights (SoftMax)	3×1	0	TD_2
Fused (Weighted sum)	32	0	Stacked + SoftMax
Output	1	33	Fused
Total trainable parameters	9,903,677		

**Table 3:** Minimum-maximum accuracy ranges across selected slices from segments 4 to 12.

Orientation	Classification Task	Hilbert-Based	Raster Row
Coronal	AD vs. CN	84.6–93.8%	50.7–92.1%
	AD vs. MCI	87.7–91.1%	80.9–87.7%
	MCI vs. CN	81.9–87.3%	72.2–80.2%
Axial	AD vs. CN	89–93.4%	85–90.3%
	AD vs. MCI	86.8–92.8%	76.2–86%
	MCI vs. CN	78.9–86.9%	74.3–80.2%
Sagittal	AD vs. CN	89–92.7%	50.7–91%
	AD vs. MCI	84.3–91.9%	77.4–85.5%
	MCI vs. CN	79.3–86.5%	73.8–81%

**Table 4:** Performance comparison between the proposed model and the model in [20] for AD vs. MCI.

Orientation	Model	Metrics	Seg4	Seg5	Seg6	Seg7	Seg8	Seg9	Seg10	Seg11	Seg12
Axial	[20]	Accuracy	76.6%	83.4%	88.1%	88.9%	81.3%	83.8%	80.0%	81.3%	77.4%
		Precision	80.9%	78.9%	87.0%	88.5%	84.8%	87.9%	74.6%	76.3%	75.4%
		Sensitivity	67.3%	89.4%	88.5%	88.5%	74.3%	77.0%	88.5%	88.5%	78.8%
		F1 Score	73.4%	83.8%	87.7%	88.5%	79.2%	82.1%	81.0%	82.0%	77.1%
		Specificity	85.2%	77.9%	87.7%	89.3%	87.7%	90.2%	72.1%	74.6%	76.2%
		AUC	84.6%	93.2%	94.6%	94.0%	91.8%	93.2%	89.0%	92.3%	82.4%
	Proposed	Accuracy	91.5%	88.9%	93.2%	92.8%	91.9%	92.8%	92.3%	89.4%	88.1%
		Precision	98.9%	93.9%	95.3%	94.4%	89.8%	92.9%	95.2%	92.3%	95.7%
		Sensitivity	83.2%	82.3%	90.3%	90.3%	93.8%	92.0%	88.5%	85.0%	78.8%
		F1 Score	90.4%	87.7%	92.7%	92.3%	91.8%	92.4%	91.7%	88.5%	86.4%
		Specificity	95.2%	96.1%	97.4%	97.7%	97.0%	97.5%	97.8%	95.4%	91.4%
		AUC	99.2%	95.1%	95.9%	95.1%	90.2%	93.4%	95.9%	93.4%	96.7%
Sagittal	[20]	Accuracy	86.0%	87.5%	88.1%	90.6%	85.1%	84.7%	89.4%	91.5%	90.6%
		Precision	81.7%	88.3%	87.0%	88.2%	81.5%	86.7%	82.8%	88.4%	91.0%
		Sensitivity	91.2%	85.0%	88.5%	92.9%	89.4%	80.5%	98.2%	94.7%	89.4%
		F1 Score	86.2%	86.6%	87.7%	90.5%	85.2%	83.5%	89.9%	91.5%	90.2%
		Specificity	81.1%	89.8%	87.7%	88.5%	81.1%	88.5%	81.1%	88.5%	91.8%
		AUC	90.7%	93.4%	93.1%	93.6%	93.7%	92.3%	97.5%	94.2%	95.9%
	Proposed	Accuracy	86.4%	91.9%	94.0%	92.3%	90.2%	86.4%	94.5%	92.8%	90.6%
		Precision	84.0%	94.3%	95.4%	92.0%	92.5%	86.5%	94.6%	91.4%	98.9%
		Sensitivity	88.5%	88.5%	92.0%	92.0%	86.7%	85.0%	93.8%	93.8%	81.4%
		F1 Score	86.2%	91.3%	93.7%	92.0%	89.5%	85.7%	94.2%	92.6%	89.3%
		Specificity	95.6%	96.5%	97.5%	97.6%	94.6%	93.6%	98.3%	98.5%	95.6%
		AUC	84.4%	95.1%	95.9%	92.6%	93.4%	87.7%	95.1%	91.8%	99.2%
Coronal	[20]	Accuracy	90.2%	87.7%	88.5%	92.3%	69.4%	90.6%	87.2%	86.0%	74.9%
		Precision	90.9%	85.6%	95.7%	92.8%	65.0%	88.9%	88.1%	87.0%	76.5%
		Sensitivity	88.5%	89.4%	79.6%	91.2%	78.8%	92.0%	85.0%	83.2%	69.0%
		F1 Score	89.7%	87.4%	87.0%	92.0%	71.2%	90.4%	86.5%	85.1%	72.6%
		Specificity	91.8%	86.1%	96.7%	93.4%	60.7%	89.3%	89.3%	88.5%	80.3%
		AUC	94.6%	92.2%	95.6%	96.4%	79.3%	95.4%	92.3%	92.9%	82.5%
	Proposed	Accuracy	93.2%	90.2%	89.4%	92.3%	90.6%	91.5%	92.3%	90.2%	89.8%
		Precision	98.0%	88.8%	90.7%	89.9%	96.9%	90.4%	90.6%	92.5%	96.8%
		Sensitivity	87.6%	91.2%	86.7%	94.7%	83.2%	92.0%	93.8%	86.7%	81.4%
		F1 Score	92.5%	90.0%	88.7%	92.2%	89.5%	91.2%	92.2%	89.5%	88.5%
		Specificity	96.0%	95.1%	94.3%	98.2%	95.6%	97.4%	97.7%	97.1%	93.4%
		AUC	98.4%	89.3%	91.8%	90.2%	97.5%	91.0%	91.0%	93.4%	97.5%

**Table 5:** Performance comparison between the proposed model and the model in [20] for MCI vs. CN.

Orientation	Model	Metrics	Seg4	Seg5	Seg6	Seg7	Seg8	Seg9	Seg10	Seg11	Seg12
Coronal	[20]	Accuracy	74.3%	81.4%	80.6%	77.2%	89.9%	83.5%	85.2%	76.8%	75.5%
		Precision	78.0%	84.2%	79.2%	87.8%	89.5%	82.2%	79.6%	71.6%	78.6%
		Sensitivity	69.7%	78.7%	84.4%	64.8%	91.0%	86.9%	95.9%	91.0%	72.1%
		F1 Score	73.6%	81.4%	81.7%	74.5%	90.2%	84.5%	87.0%	80.1%	75.2%
		Specificity	79.1%	84.3%	76.5%	90.4%	88.7%	80.0%	73.9%	61.7%	79.1%
		AUC	82.9%	91.5%	91.4%	86.7%	95.8%	92.2%	95.7%	87.5%	84.3%
	Proposed	Accuracy	85.7%	88.2%	86.9%	88.6%	89.9%	91.1%	84.4%	84.0%	87.3%
		Precision	87.9%	83.1%	82.7%	91.3%	90.2%	90.4%	81.0%	81.3%	84.8%
		Sensitivity	83.6%	96.7%	94.3%	86.1%	90.2%	92.6%	91.0%	89.3%	91.8%
		F1 Score	85.7%	89.4%	88.1%	88.6%	90.2%	91.5%	85.7%	85.2%	88.2%
		Specificity	94.1%	94.9%	93.7%	96.0%	96.0%	96.5%	93.1%	91.8%	94.3%
		AUC	87.8%	79.1%	79.1%	91.3%	89.6%	89.6%	77.4%	78.3%	82.6%
Sagittal	[20]	Accuracy	86.9%	81.4%	85.7%	81.4%	82.7%	84.8%	84.8%	86.5%	80.6%
		Precision	85.8%	81.5%	85.5%	85.5%	80.5%	80.7%	93.9%	88.8%	83.9%
		Sensitivity	89.3%	82.8%	86.9%	77.0%	87.7%	92.6%	75.4%	84.4%	77.0%
		F1 Score	87.6%	82.1%	86.2%	81.0%	83.9%	86.3%	83.6%	86.6%	80.3%
		Specificity	84.3%	80.0%	84.3%	86.1%	77.4%	76.5%	94.8%	88.7%	84.3%
		AUC	92.6%	91.2%	93.2%	89.5%	88.4%	92.4%	92.7%	92.5%	89.5%
	Proposed	Accuracy	92.0%	81.4%	88.6%	93.2%	88.2%	86.1%	89.9%	90.3%	87.3%
		Precision	94.0%	92.4%	85.7%	92.1%	87.9%	89.4%	87.7%	89.0%	83.3%
		Sensitivity	90.2%	69.7%	93.4%	95.1%	89.3%	82.8%	93.4%	92.6%	94.3%
		F1 Score	92.1%	79.4%	89.4%	93.5%	88.6%	86.0%	90.5%	90.8%	88.5%
		Specificity	97.0%	93.2%	95.3%	98.0%	93.1%	95.1%	96.3%	93.7%	94.3%
		AUC	93.9%	93.9%	83.5%	91.3%	87.0%	89.6%	86.1%	87.8%	80.0%
Axial	[20]	Accuracy	86.5%	84.4%	85.2%	82.7%	85.2%	86.9%	82.7%	70.5%	75.5%
		Precision	89.5%	84.6%	92.2%	77.9%	84.3%	85.8%	79.1%	77.7%	76.2%
		Sensitivity	83.6%	85.2%	77.9%	92.6%	87.7%	89.3%	90.2%	59.8%	76.2%
		F1 Score	86.4%	84.9%	84.4%	84.6%	85.9%	87.6%	84.3%	67.6%	76.2%
		Specificity	89.6%	83.5%	93.0%	72.2%	82.6%	84.3%	74.8%	81.7%	74.8%
		AUC	93.8%	91.4%	91.9%	90.4%	90.8%	93.9%	90.2%	82.6%	82.7%
	Proposed	Accuracy	89.5%	87.8%	92.4%	85.7%	86.5%	87.3%	88.2%	88.6%	78.9%
		Precision	88.8%	85.0%	91.3%	84.9%	84.6%	84.3%	85.6%	87.4%	79.5%
		Sensitivity	91.0%	92.6%	94.3%	87.7%	90.2%	92.6%	92.6%	91.0%	79.5%
		F1 Score	89.9%	88.6%	92.7%	86.3%	87.3%	88.3%	89.0%	89.2%	79.5%
		Specificity	95.3%	94.3%	97.2%	92.6%	92.7%	93.7%	95.1%	95.8%	89.7%
		AUC	87.8%	82.6%	90.4%	83.5%	82.6%	81.7%	83.5%	86.1%	78.3%

**Table 6:** Performance comparison between the proposed model and the model in [20] for AD vs. CN.

Orientation	Model	Metrics	Seg4	Seg5	Seg6	Seg7	Seg8	Seg9	Seg10	Seg11	Seg12
Coronal	[20]	Accuracy	89.4%	94.3%	95.2%	91.2%	95.6%	94.3%	96.9%	89.0%	92.1%
		Precision	87.3%	92.3%	91.7%	91.1%	93.2%	95.4%	97.3%	88.5%	88.5%
		Sensitivity	92.0%	96.4%	99.1%	91.1%	98.2%	92.9%	96.4%	89.3%	96.4%
		F1 Score	89.6%	94.3%	95.3%	91.1%	95.7%	94.1%	96.9%	88.9%	92.3%
		Specificity	87.0%	92.2%	91.3%	91.3%	93.0%	95.7%	97.4%	88.7%	87.8%
		AUC	95.6%	98.1%	99.2%	96.2%	99.2%	98.5%	99.5%	94.9%	97.1%
	Proposed	Accuracy	95.2%	95.2%	96.0%	96.9%	96.9%	96.5%	96.5%	96.0%	93.4%
		Precision	94.7%	96.3%	98.1%	97.3%	95.7%	99.1%	94.1%	95.6%	95.3%
		Sensitivity	95.5%	93.8%	93.8%	96.4%	98.2%	93.8%	99.1%	96.4%	91.1%
		F1 Score	95.1%	95.0%	95.9%	96.9%	96.9%	96.3%	96.5%	96.0%	93.2%
		Specificity	99.1%	99.1%	99.3%	99.5%	98.9%	99.5%	99.5%	99.5%	98.7%
		AUC	94.8%	96.5%	98.3%	97.4%	95.7%	99.1%	93.9%	95.7%	95.7%
Sagittal	[20]	Accuracy	92.5%	89.4%	96.0%	94.3%	84.6%	91.2%	96.9%	95.6%	89.0%
		Precision	91.3%	87.9%	99.0%	93.8%	87.4%	88.3%	96.5%	94.7%	88.5%
		Sensitivity	93.8%	91.1%	92.9%	94.6%	80.4%	94.6%	97.3%	96.4%	89.3%
		F1 Score	92.5%	89.5%	95.9%	94.2%	83.7%	91.4%	96.9%	95.6%	88.9%
		Specificity	91.3%	87.8%	99.1%	93.9%	88.7%	87.8%	96.5%	94.8%	88.7%
		AUC	97.2%	96.5%	97.3%	98.2%	92.0%	97.5%	98.6%	97.9%	96.4%
	Proposed	Accuracy	96.5%	96.5%	95.6%	96.0%	89.0%	95.6%	98.2%	97.8%	91.2%
		Precision	95.6%	94.8%	97.2%	98.1%	94.8%	99.0%	98.2%	99.1%	90.4%
		Sensitivity	97.3%	98.2%	93.8%	93.8%	82.1%	92.0%	98.2%	96.4%	92.0%
		F1 Score	96.5%	96.5%	95.5%	95.9%	88.0%	95.4%	98.2%	97.7%	91.2%
		Specificity	99.3%	99.4%	98.9%	99.1%	97.3%	98.9%	99.8%	99.7%	97.7%
		AUC	95.7%	94.8%	97.4%	98.3%	95.7%	99.1%	98.3%	99.1%	90.4%
Axial	[20]	Accuracy	92.5%	94.7%	89.0%	92.5%	94.3%	97.4%	93.8%	93.0%	92.1%
		Precision	94.4%	92.4%	85.4%	91.3%	93.8%	96.5%	94.5%	97.1%	90.5%
		Sensitivity	90.2%	97.3%	93.8%	93.8%	94.6%	98.2%	92.9%	88.4%	93.8%
		F1 Score	92.2%	94.8%	89.4%	92.5%	94.2%	97.3%	93.7%	92.5%	92.1%
		Specificity	94.8%	92.2%	84.3%	91.3%	93.9%	96.5%	94.8%	97.4%	90.4%
		AUC	97.0%	98.7%	94.7%	94.8%	97.7%	99.6%	97.2%	98.9%	96.5%
	Proposed	Accuracy	94.7%	93.8%	96.0%	91.2%	96.0%	97.8%	96.9%	94.7%	93.4%
		Precision	95.5%	92.2%	95.6%	90.4%	92.6%	97.3%	95.7%	98.1%	92.9%
		Sensitivity	93.8%	95.5%	96.4%	92.0%	100.0%	98.2%	98.2%	91.1%	93.8%
		F1 Score	94.6%	93.9%	96.0%	91.2%	96.1%	97.8%	96.9%	94.4%	93.3%
		Specificity	98.7%	99.1%	99.5%	98.4%	99.7%	99.5%	99.7%	99.5%	98.7%
		AUC	95.7%	92.2%	95.7%	90.4%	92.2%	97.4%	95.7%	98.3%	93.0%

**Table 7:** Flops Comparison of MRI-based part and Baseline Lightweight Models.

Method	FLOPs
MobileNetV1	467,840,993
EfficientNetB0	397,007,176
MobileNetV2	301,415,457
Image-Stream	78,102,649

## Data Availability

The data presented in this study are openly available in The Image and Data Archive (IDA) at https://ida.loni.usc.edu/login.jsp?project=ADNI (accessed on 10 December 2025).
